# Supplementary figures and images for: Association between sleep duration and hypertension incidence: Systematic review and meta-analysis of cohort studies
Source: PLoS One. 2024 Jul 15;19(7):e0307120. doi: 10.1371/journal.pone.0307120 (PMC11249221; doi:10.1371/journal.pone.0307120)

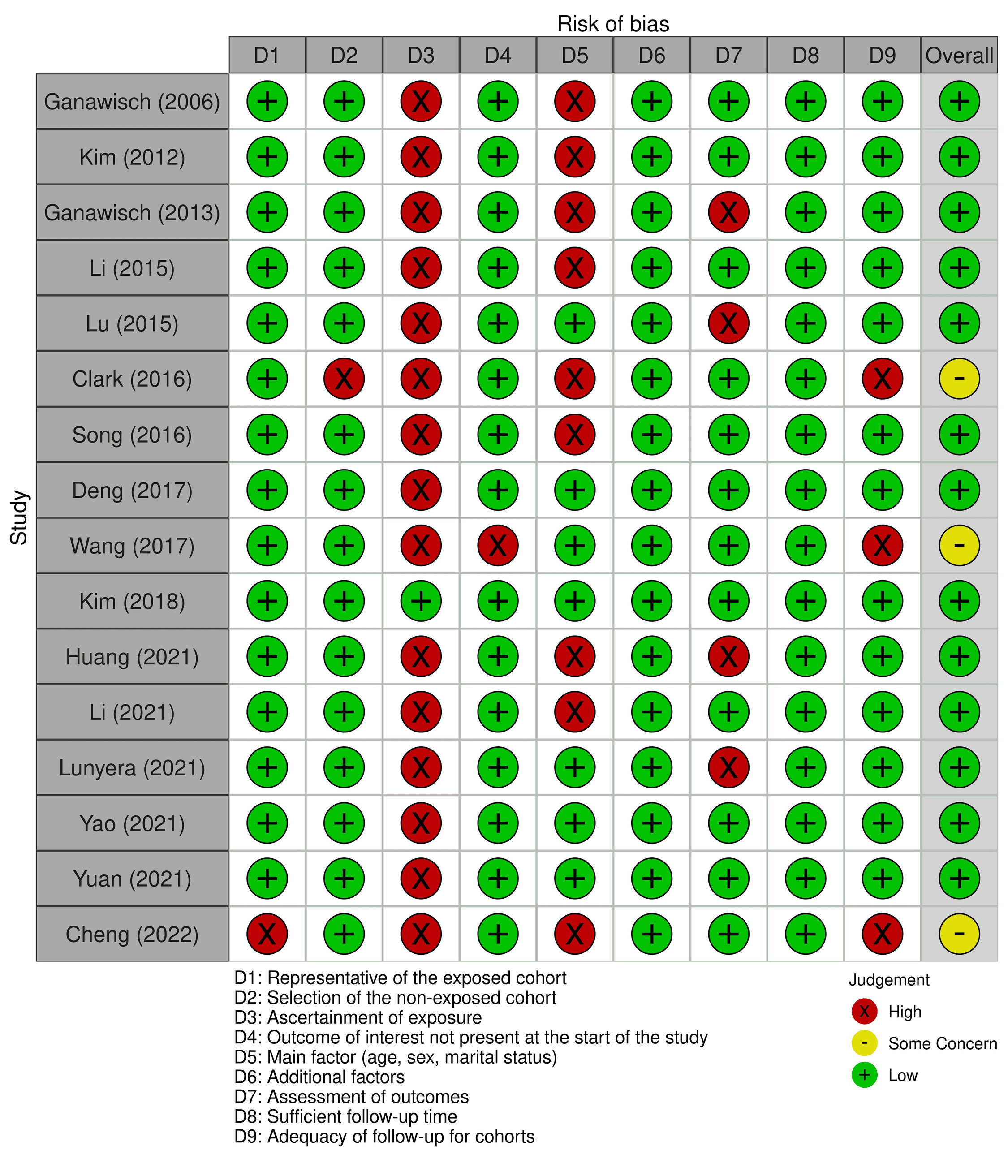

Supplement: S1 Fig — (TIF) [file pone.0307120.s001.tif]

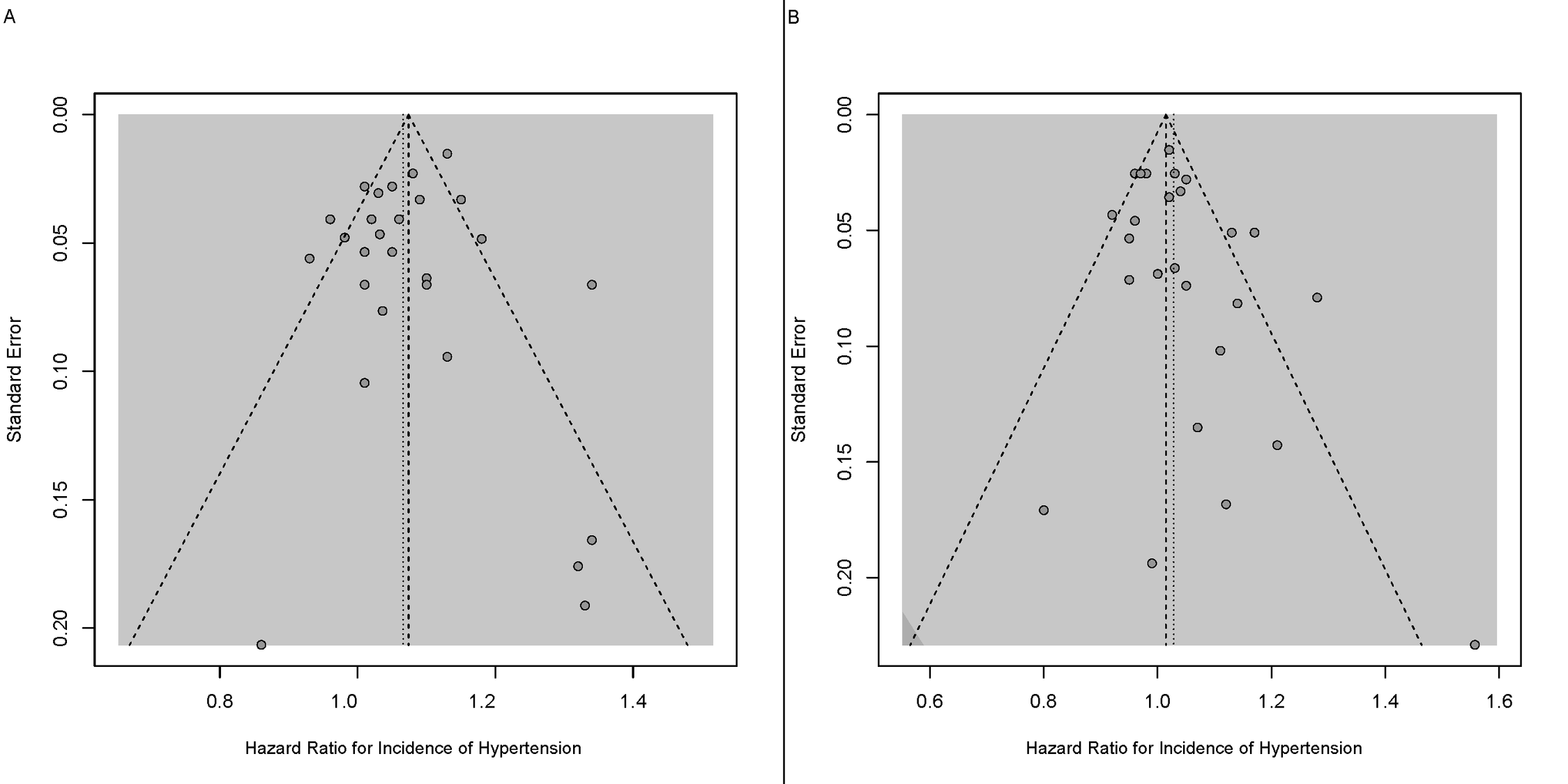

Supplement: S2 Fig — (A) short duration of sleep compared with the reference group and (B) long duration of sleep compared with the reference group. (TIF) [file pone.0307120.s002.tif]

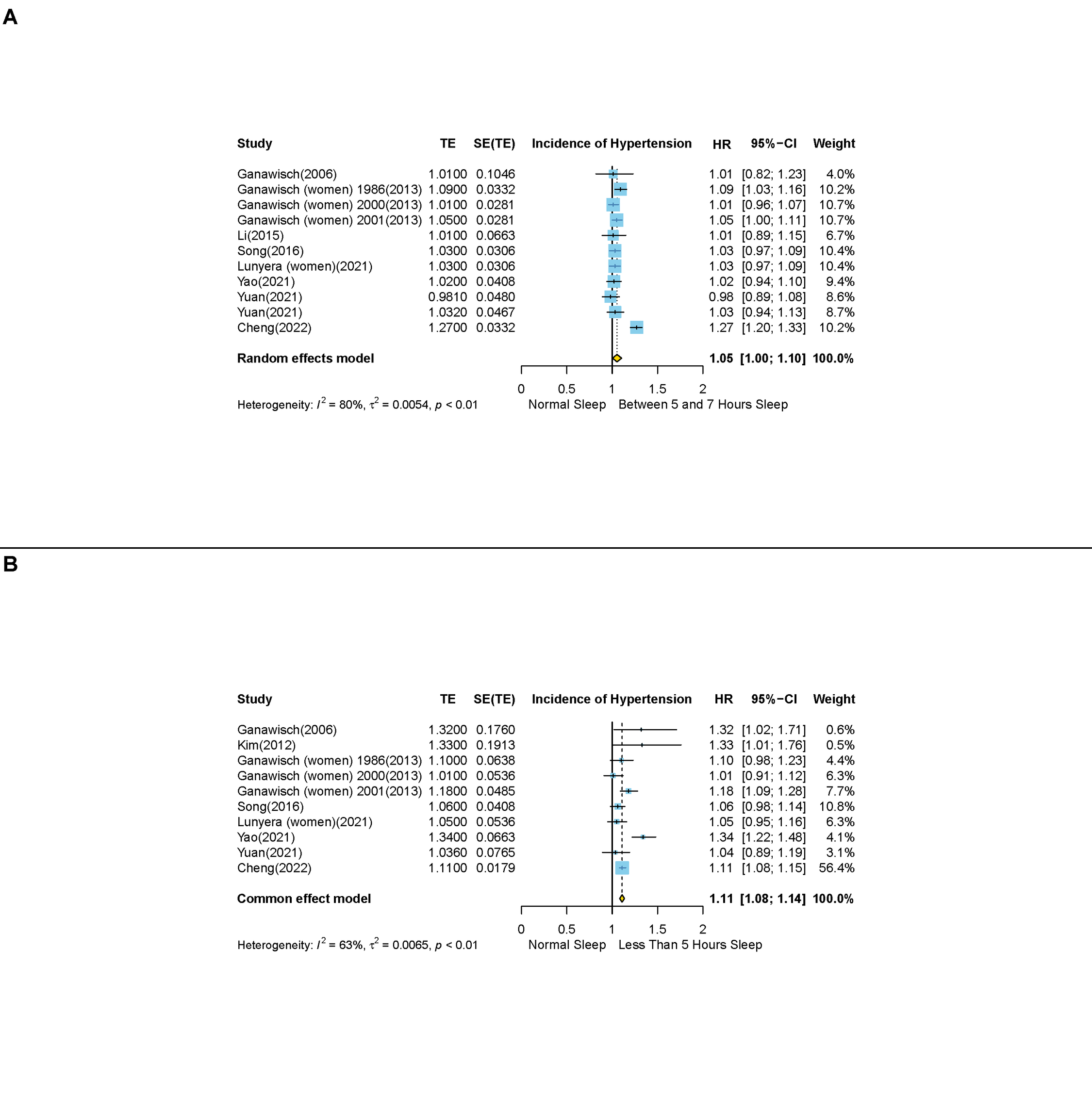

Supplement: S4 Fig — (A) 5–7 hours of sleep duration compared with the reference group and (B) less than 5 hours of sleep duration compared with the reference group. Results are expressed as Hazard ratio and 95% confidence intervals. (TIF) [file pone.0307120.s004.tif]

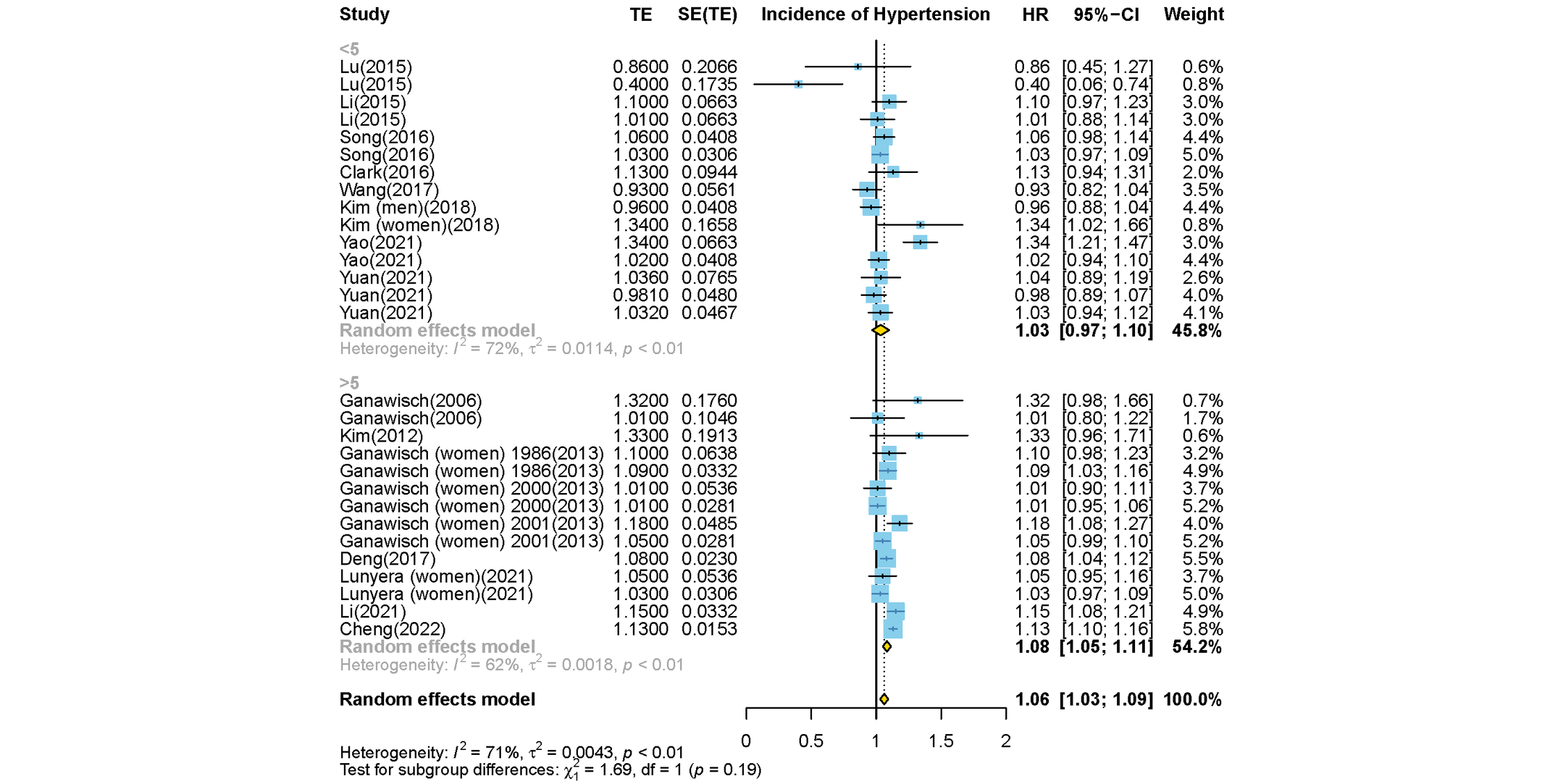

Supplement: S5 Fig — Results are expressed as Hazard ratio and 95% confidence intervals. (TIF) [file pone.0307120.s005.tif]

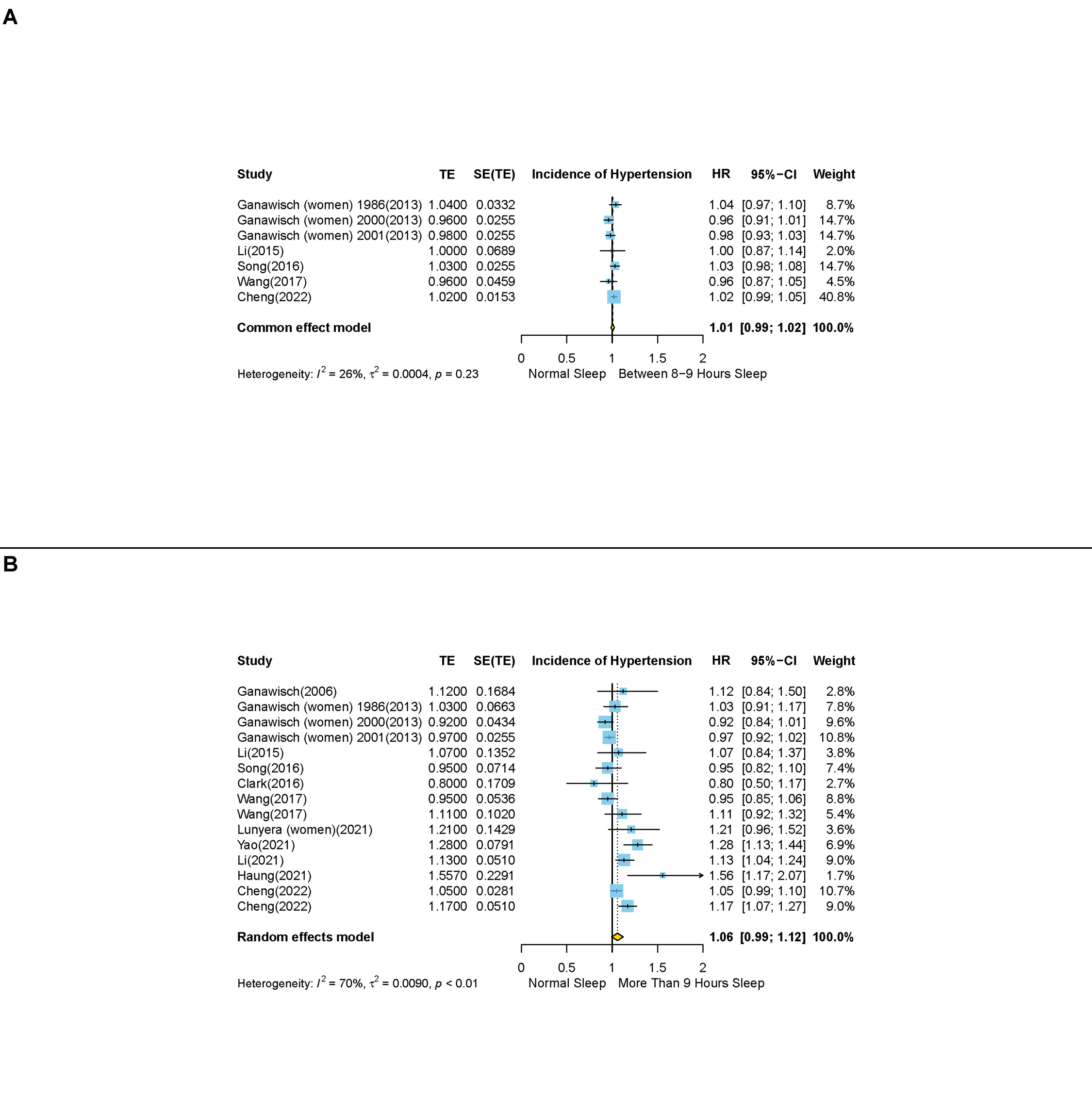

Supplement: S6 Fig — (A) 8–9 hours of sleep duration compared with the reference group and (B) more than 9 hours of sleep duration compared with the reference group. Results are expressed as Hazard ratio and 95% confidence intervals. (TIF) [file pone.0307120.s006.tif]

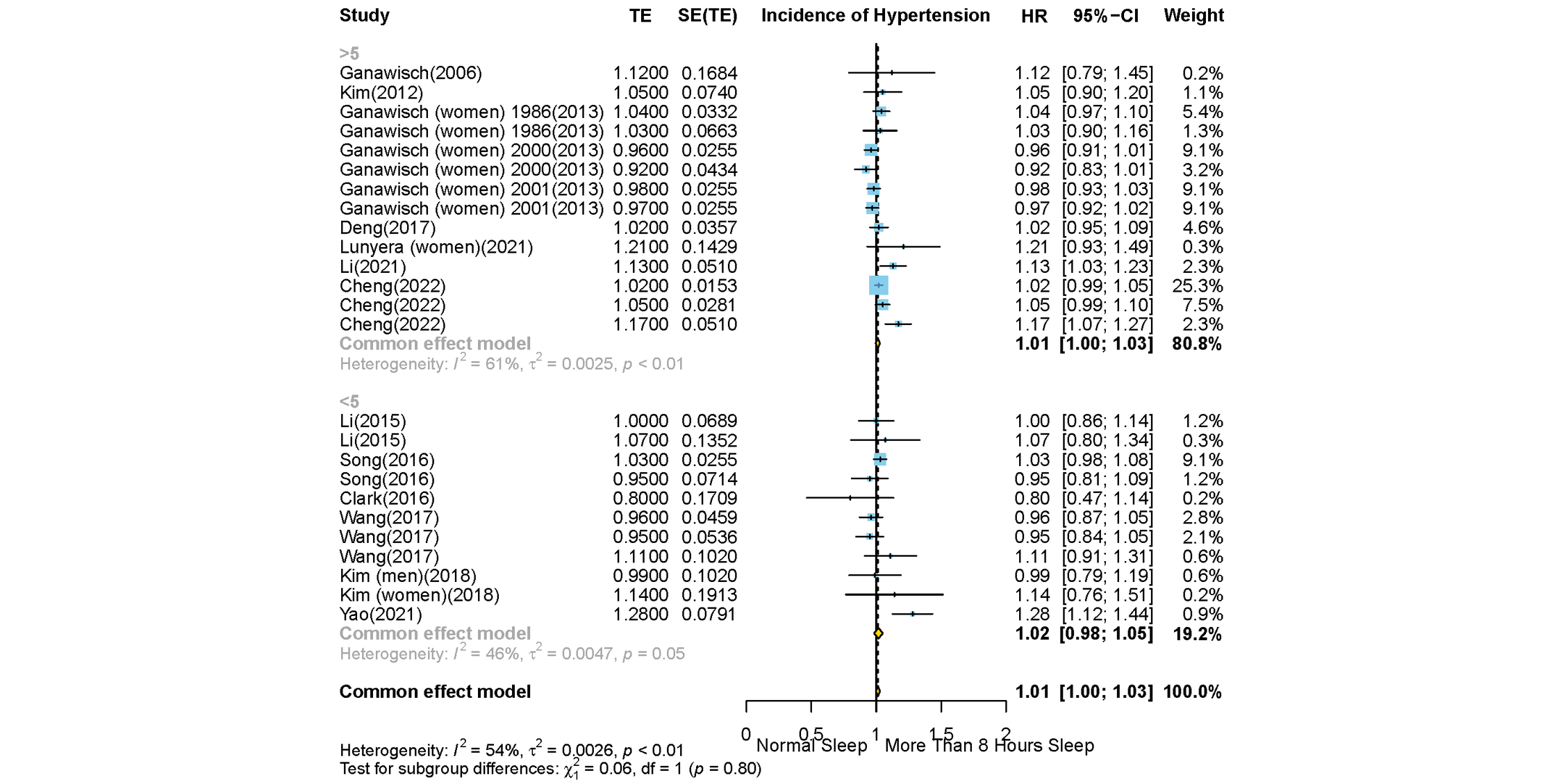

Supplement: S7 Fig — Results are expressed as Hazard ratio and 95% confidence intervals. (TIF) [file pone.0307120.s007.tif]

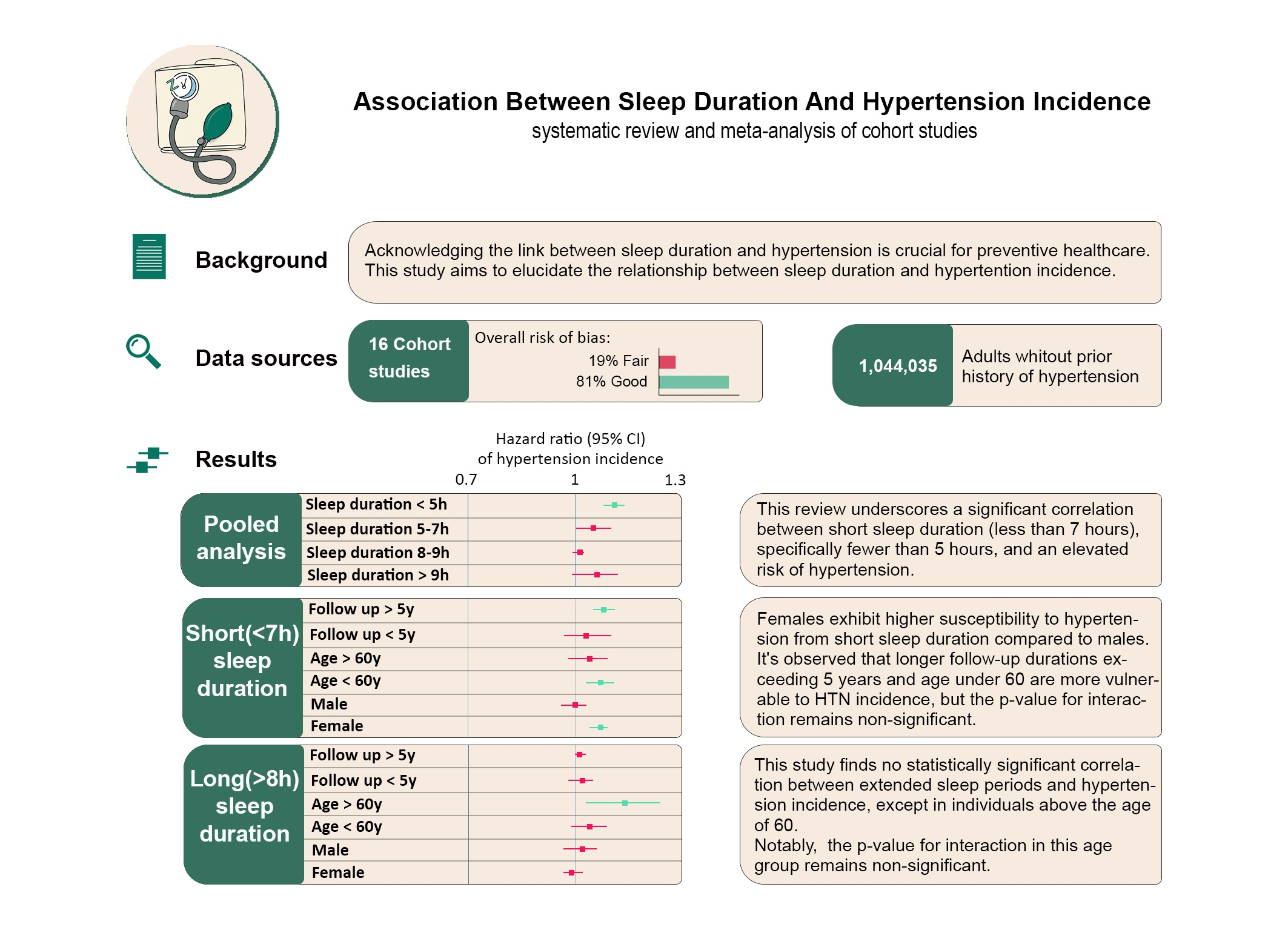

Supplement: S1 Graphical abstract — (TIF) [file pone.0307120.s011.tif]
