# Supplementary material for: Association between sleep duration and hypertension incidence: Systematic review and meta-analysis of cohort studies
Source: PLoS One. 2024 Jul 15;19(7):e0307120. doi: 10.1371/journal.pone.0307120 (PMC11249221; doi:10.1371/journal.pone.0307120)
Supplement: S2 Table — (DOCX) [file pone.0307120.s009.docx]

| **Sleep** |
| --- |
| Sleep, Circadian Rhythms, Habit* AND Sleep*, Deprivation AND Sleep, Sleep Deprivation, Sleep Insufficien*, Insufficien* AND Sleep, Inadequate Sleep, Sleep AND Inadequate, Sleep Fragmentation, Fragmentation AND Sleep, Sleep Debt, Duration AND Sleep, Sleep Time, Sleep Qualit*, Qualit* AND Sleep, Quantity AND Sleep, Sleep Quantit*, Hygiene AND Sleep, Sleep latenc*, Latenc* AND Sleep, Sleep Stage*, Stage* AND Sleep, Paradoxical Sleep, Sleep AND Paradoxical, atypical sleep, difficult* sleeping, Sleep disorder*, Sleep disturbance*, Sleep AND disturbance*, Sleep problem*, Sleep AND problem*, trouble sleep*, sleep symptom*, sleep pattern, insomnia, dyssomn*, sleep characteristic*, sleep impairment*, sleep behavior* |
| **Hypertension** |
| Hypertension, Blood Pressure, Blood Pressure AND High, Blood Pressures AND High, High Blood Pressure, High Blood Pressures, Hypertensive, HTN, intravascular pressure, Elevated blood pressure, Elevated blood pressures, Increased blood pressure, Increased blood pressures |
