# Supplementary material for: Association between sleep duration and hypertension incidence: Systematic review and meta-analysis of cohort studies
Source: PLoS One. 2024 Jul 15;19(7):e0307120. doi: 10.1371/journal.pone.0307120 (PMC11249221; doi:10.1371/journal.pone.0307120)
Supplement: Attachment — Submitted filename: Response to Reviewers.docx [file pone.0307120.s012.docx]

Dear editorial team

We deeply appreciate the constructive feedback provided on our article. Our authors have revised the article in accordance with the mentioned comments, resulting in much more robust manuscript. Should there be any additional concerns regarding this manuscript, please feel free to direct them to me at the following address

[hamid.r.soleimani90@gmail.com](mailto:hamid.r.soleimani90@gmail.com)

Sincerely,

Hamidreza Soleimani

**Point by point answers:**

Journal requirements

1. When submitting your revision, please ensure that your manuscript meets PLOS ONE's style requirements, including those for file naming

Answer: The article was revised based on the PLOS ONE's style requirements

2. PLOS requires an ORCID iD for the corresponding author in Editorial Manager on papers submitted after December 6th, 2016. Please ensure that you have an ORCID iD and that it is validated in Editorial Manager.

Answer: The ORCID ID of the corresponding author has been submitted.

3. We noted in your submission details that a portion of your manuscript may have been presented or published elsewhere. [The article's abstract has been submitted to the "American College of Cardiology 2024" conference; however, we are still awaiting notification regarding its acceptance. It was a conference will not constitute dual publication.] Please clarify whether this conference proceeding was peer-reviewed and formally published. If this work was previously peer-reviewed and published, in the cover letter please provide the reason that this work does not constitute dual publication and should be included in the current manuscript.

Answer: We have previously mentioned that the abstract of the article was submitted for presentation in "American College of Cardiology 2024" (ACC 2024) conference with previous knowledge that this presentation does not constitute dual publication. The presentation was accepted by ACC 2024 and we mentioned in the conference that the article is currently under review for publication in PLOs ONE journal.

4. We note that you have indicated that there are restrictions to data sharing for this study. PLOS only allows data to be available upon request if there are legal or ethical restrictions on sharing data publicly. If there are no restrictions, please upload the minimal anonymized data set necessary to replicate your study findings to a stable, public repository and provide us with the relevant URLs, DOIs, or accession numbers.

Answer: We have submitted the data of the included studies as supplementary information file.

5. In the online submission form, you indicated that [The data underlying the results presented in the study are available on request from the author and were gathered from the published studies included in our meta-analysis.]. All PLOS journals now require all data underlying the findings described in their manuscript to be freely available to other researchers, either 1. In a public repository, 2. Within the manuscript itself, or 3. Uploaded as supplementary information.

Answer: We have provided the data of the included studies as supplementary information file.

Reviewers' comments

Reviewer #1:

1. Title:

The title should be more specific and concise, indicating the focus of the study. For example, "Association Between Sleep Duration and Hypertension Incidence: A Meta-Analysis."

Answer: Thank you so much for your valuable comment. We approved the title

2. Abstract:

• Method: Specify the statistical methods used for pooling hazard ratios and risk ratios (e.g., DerSimonian and Laird random-effects model).

Answer: Thank you so much for your true comment. The text has been changed: line 41-42

• Results: Line 43 "Ranging from 2.4 to 18 years" might be better as "ranging from 2.4 to 18 years of follow-up duration."

Answer: Thank you so much for your insightful comment. The text has been changed: line 43

• Conclusion: The conclusion should summarize the main findings of the study and their implications for clinical practice or further research.

Answer: Your comment has been invaluable. The text has been changed: line 50-53

3. Introduction:

• When discussing the association between sleep duration and HTN, it would be helpful to briefly mention some of the conflicting findings in the existing literature to highlight the need for the current study.

Answer: Thank you so much for your valuable comment. The text has been changed: line 71-78

• The objectives of the study are clearly stated, but it might be beneficial to explicitly mention the primary aim of the meta-analysis (e.g., to quantify the association between sleep duration and HTN incidence) before discussing stratification by age and sex.

Answer: Thank you for sharing your expertise. The text has been changed: line 81

• Consider including a brief transition at the end of the introduction to smoothly lead into the methods section, such as mentioning the need for a systematic review and meta-analysis to address the existing uncertainties.

Answer: Thank you so much for your insightful comment. The text has been changed: line 71-78

• Line 56-57: Consider rephrasing "With a worldwide prevalence estimated at 34% in men and 32% in women" to specify that these estimates pertain to the prevalence of hypertension.

Answer: Thank you so much for your true comment. The text has been changed: line 59-60

4. Methods:

• Registration of Protocol: Specify whether the protocol was registered before the initiation of the study. Also, mention the publication date of the protocol if applicable.

Answer: Thank you so much for your true comment. The text has been changed: line 88

• Clarify the rationale behind excluding studies with a sample size of less than 30 people.

Answer: Thank you so much for your comment. The text has been changed: line 96

• Line 127-129: Please provide references for the definitions of sleep duration utilized in the study.

Answer: Thank you so much for your valuable comment. The text has been changed: line 140-141

• When discussing outcomes, providing examples of covariates used for adjustment could enhance understanding. Additionally, specify how missing data were handled during outcome extraction.

Answer: Thank you so much for your true comment. The text has been changed: line 143 and 162-165

5. Discussion:

• Line 226-227: The statement "To our knowledge, this meta-analysis has been the most comprehensive study that exploring the relationship between sleep duration and HTN incidence" could be revised for clarity. For instance, you could say, "This meta-analysis represents a comprehensive investigation into the relationship between sleep duration and HTN incidence, encompassing various definitions of short and long sleep durations, as well as follow-up durations.

Answer: Thank you so much for the guidance. The text has been changed: line 243-245

• While the discussion references previous meta-analyses and systematic reviews on the topic, it could be strengthened by providing a more detailed comparison of the current findings with those of previous studies. This would help contextualize the significance of the current study and highlight any novel contributions.

Answer: Thank you so much for your constructive comment. The text has been changed: line 248-258

• When discussing the association between short sleep duration and HTN incidence, consider elaborating on the potential clinical implications of these findings. For example, you could discuss the importance of early detection and intervention for individuals with short sleep duration to reduce their risk of developing HTN.

Answer: Thank you so much for your thoughtful comment. The text has been changed: line 299-303

• The limitations of the study are appropriately acknowledged in the discussion. To strengthen this section, consider providing a more detailed discussion of how these limitations may have influenced the results and interpretations. Additionally, discuss potential strategies to address these limitations in future research.

Answer: Thank you for your guidance. The text has been changed: line 319-326

• The conclusion effectively summarizes the key findings of the study and emphasizes the implications for clinical practice and future research. To enhance the conclusion, consider reinforcing the main implications of the findings and providing recommendations for future research directions or public health interventions.

Answer: Thank you so much for your true comment. The text has been changed: line 330-332

Reviewer #2:

1. Abstract:

* Please highlight the comparison group in your methods.

Answer: Thank you so much for your valuable comment. The text has been changed: line 40

* Try to use "risk" before using "incidence" of HTN throughout the manuscript.

Answer: Thank you so much for your true comment. The text has been changed.

* Use pooled "RR" instead of pooled "HR" throughout the manuscript, as you are combining different types of association measures.

Answer: All of the included studies in this article used HR as their effect size for assessment, except the study conducted by Haung et al. (<https://www.ncbi.nlm.nih.gov/pmc/articles/PMC8517638/> ). Consequently, we kept HR due to its ability to consider both the incidence and the timing beside mitigating the potential confounders, therefore providing stronger support for causal relationship between different sleep durations and HTN incidence.

2. Introduction:

* Please state the clinical impressions of your study in the last paragraph of the Introduction. What problems remain unanswered? What are questions responding to?

Answer: Thank you so much for your valuable comment. The text has been changed: line 71-78

3. Methods:

* Please check for consistency with the PRISMA guideline!

Answer: Thank you so much for your insightful comment. We rechecked the consistency of the article with PRISMA guidelines and presented the results in S1 table.

* Explain your methods in more detail! How many reviewers contributed to different processes? You should also cite references regarding heterogeneity, Egger's test, etc.

Answer: Thank you so much for your guidance. The method section has been enhanced.

* The NOS assessed quality or risk of bias? These are different!

Answer: Thank you for your true comment. The text has been changed: line 116-117

4. Results:

* Table 1 is not observed completely!

Answer: Thank you for your true comment. The Table 1 has been corrected.
